# Association between Oxidative Stress Parameters and Hematological Indices in Breast Cancer Patients

**DOI:** 10.1155/2022/1459410

**Published:** 2022-10-03

**Authors:** Hiva Danesh, Nasrin Ziamajidi, Seyed Alireza Mesbah-Namin, Nahid Nafisi, Roghayeh Abbasalipourkabir

**Affiliations:** ^1^Department of Clinical Biochemistry, School of Medicine, Hamadan University of Medical Sciences, Hamadan, Iran; ^2^Department of Clinical Biochemistry, Faculty of Medical Sciences, Tarbiat Modares University, Tehran, Iran; ^3^Department of General Surgery, Hazrat-e Rasool General Hospital, Iran University of Medical Sciences, Tehran, Iran

## Abstract

**Background:**

Breast cancer is one of the leading causes of death in women worldwide. This causes an increase in free radicals, resulting in oxidative stress. The aim of this study was to determine the effect of breast cancer on oxidative stress and its relationship with hematological indices.

**Methods:**

This case-control study included 43 women with breast cancer and 37 age-matched healthy controls. Oxidative stress and its correlation with hematological profiles over seven months were evaluated. Finally, the data were compared between the two groups using the *t*-test and Pearson's test, and the results were analyzed using the SPSS 24 software.

**Results:**

The results revealed that patients with breast cancer had significantly increased hemoglobin (HB), hematocrit (HCT), mean corpuscular volume (MCV), and mean corpuscular hemoglobin (MCH) levels compared with healthy subjects (*p* < 0.05). In addition, oxidative stress parameters, such as superoxide dismutase (SOD), catalase (CAT), total oxidant status (TOS), and total antioxidant capacity (TAC), were significantly elevated. Glutathione peroxidase (GPX) and malondialdehyde (MDA) were significantly lower in patients with breast cancer than in the control group (*p* < 0.05). Statistical significance in hematological indices showed a positive or negative correlation with oxidative stress parameters.

**Conclusion:**

Women with breast cancer showed a deranged complete blood count (CBC) pattern compared to healthy individuals.

## 1. Introduction

Breast cancer is the most common cancer and the second leading cause of death among women in the United States [1]. According to the World Health Organization (WHO), breast cancer is one of the most diagnosed cancers among women and its incidence is increasing every day [2]. According to the latest statistics from the Cancer Research Center in Iran, approximately 8500 new cases of breast cancer are registered annually in the country, and 1400 cases cause death [3]. Therefore, the earlier this cancer is diagnosed, the easier and more successful the treatment. The etiology of breast cancer includes age, late menopause, contraceptive use, hormone therapy, family history, and obesity. [4]. These risk factors exert their effects through oxidative stress [5].

Oxidative stress is an imbalance between oxidants (free radicals) and antioxidants [6]. This disorder results in an increase in free radicals, an imbalance between the production and elimination of active species in the body, and a decrease in the strength of antioxidant defense system [7]. Oxidative stress is evidenced by changes in antioxidant status and altered activities of cellular enzymes, such as superoxide dismutase (SOD), glutathione peroxidase (GPX), and catalase (CAT) [8]. We can assess the oxidative stress by assessment of some indices such as malondialdehyde (MDA), total oxidant status (TOS), and total antioxidant capacity (TAC) in the patient's serum. Many cellular processes, including metabolism, signaling pathways, regulatory pathways for gene expression, cell proliferation, and programmed cell death, are affected by oxidative stress [9]. Free radicals increase to alter the structure and function of the body's major biological molecules, including proteins, lipids, and nucleic acids, ultimately leading to tissue damage [10, 11].

Oxidative stress may affect the functions of blood cells, coagulation system, and lipid profile, resulting in acute and chronic infections, anemia, and hypercoagulability [7]. The complete blood count (CBC) reflects the cellular immune response in a cancer patient and any changes in hematological parameters influence cancer progression [12]. Recently, definite indices from CBC have been shown to be valuable in predicting outcomes in patients with breast cancer. Therefore, it is important to study CBC in patients with breast carcinoma.

This study was performed twice. First, we determined whether breast cancer patients have higher oxidative stress, and assessed the relationship between oxidative stress and changes in hematological indices.

## 2. Material and Methods

### 2.1. Study Site

The present case-control study was conducted for 7 months from May to Jan 2022. This study was carried out by the Department of Clinical Biochemistry at Hamadan University of Medical Sciences with the participation of Khatam Al-Anbia Hospital in Tehran, Iran.

### 2.2. Study Population

The study population comprised thirty-seven healthy subject and forty-three women with breast cancer (28-80 years), who were referred to Khatam Al-Anbia Hospital. A volunteer without cancer or any systemic disease at the same age was enrolled as a control group. The exclusion criteria included breast removal surgery, chemotherapy, hormone therapy, radiation therapy, recurrent specimens, and history of tumors in other tissues and organs. After obtaining a consent form and completing a demographic questionnaire from the participants, 5 mL blood samples were taken by standard intravenous sampling method after 8 hours of fasting and collected in a tube without anticoagulant to obtain the serum. Immediately after sampling, the tubes were transferred to a medical center laboratory. Serum was prepared by centrifugation at 3000 rpm for 10 min at 4°C and stored at −20°C until assayed.

### 2.3. Hematological Analysis

The hematological indices including the total number of white blood cells (WBC), total red blood cells (RBC) count, hemoglobin content (Hb), hematocrit (HCT), mean corpuscular volume (MCV), mean corpuscular hemoglobin (MCH), MCH concentration (MCHC), and platelets (PLT) were analyzed using an automated hematology analyzer (Sysmex KX-21 N, Germany).

### 2.4. Biochemical Analysis

After 12 h of fasting, 5 cc blood samples were collected and serum was isolated by centrifugation at 2400 rpm for 15 min. Serum was used to evaluate biochemical parameters, including aspartate aminotransferase (AST), and alanine aminotransferase (ALT) levels, using colorimetric methods (Pars Azmoon, Tehran, Iran) on a BIOLIS24i Premium autoanalyzer (Tokyo Boeki Machinery Ltd., Japan).

### 2.5. Analysis of Oxidative Stress

Oxidative stress indices including MDA, TAC, TOS, CAT, GPX, and SOD, were evaluated using commercial kits, according to the manufacturer's instructions. Plasma was used to measure MDA, TOS, TAC, CAT, SOD, and GPx activities. The oxidative degradation of lipids in the presence of free radicals is known as lipid peroxidation.

### 2.6. SOD Assay

The SOD activity was determined using a commercial kit (SOD activity Kiazist, Iran) according to the manufacturer's instructions. We used a calorimetric method to measure SOD activity. This method is based on the ability of Mn-SOD to inhibit the conversion of resazurin to resorufin accompanied by the reduction of superoxide radicals produced by the xanthine/xanthine oxidase system. Finally, the absorbance was read at a wavelength-570-520 nm (recommended at 570 nm).

### 2.7. CAT Assay

In this experiment, catalase had peroxidative activity in the presence of methanol, and then it was stopped in the presence of its inhibitor, and the formaldehyde produced, reacted with Purpald and produced a purple color. This dye absorbs light at a wavelength of 540 nm.

### 2.8. GPx Assay

GPx activity was measured using a commercial kit (GPx activity Kiazist, Iran) according to the manufacturer's instructions. In this kit, the coupling reaction is performed along with the enzyme glutathione reductase and its coenzyme NADPH. This method is based on the reduction of hydrogen peroxide to water accompanied by the oxidation of glutathione. The absorbance was immediately read at 340 nm for 5 min and every one minute in the kinetic mode.

### 2.9. Lipid Peroxidation Assay (MDA)

MDA, one of the most important end products of lipid peroxidation, was measured a commercial kit (MDA concentration Kiazist; Iran), according to the manufacturer's instructions. In this experiment, MDA formed a complex with thiobarbituric acid and was absorbed at a wavelength of 532 nm.

### 2.10. Total Oxidant Status (TOS)

The ability of the samples to change ferrous ions (Fe III) to ferric ions (Fe II) and produce color in the presence of chromogen was determined by measuring the total oxidant status (TOS). This color had a wavelength of 550-580 nm. The reaction between ferric ions and xylenol orange forms a colored complex. The assay was calibrated using H_2_O_2_. The amount of absorption was directly related to the amount of oxidant, and the standard curve was drawn in the presence of 2O2H.

### 2.11. Total Antioxidant Capacity (TAC)

In this experiment, cupric (Cu+2) is reduced to cupro (Cu+1) in the presence of antioxidants and produced color in the presence of a chromogen. This color was absorbed at a wavelength of 450 nm. The amount of absorption is directly related to the amount of antioxidants.

### 2.12. Data Analysis

Statistical analyses were performed using SPSS version 24. Quantitative results were reported based on the mean and standard deviation. Independent *t*-tests and Pearson's correlation analyses were used to determine the mean differences and correlation between oxidative stress and hematological parameters, respectively. The *p* value ≤ .05 was considered significant.

## 3. Results

### 3.1. Clinical Data

Eighty participants were enrolled in the study, including 43 subjects with breast cancer (cases) and 37 subjects without breast cancer (controls). The mean ages for cases and controls were in 53.79 ± 11.95 years and 53.75 ± 12.92 years, respectively. Women with breast cancer were divided into two groups of patients with BMI less than 30 kg/m^2^ and more than 30 kg/m^2^ (Table 1), and the immunohistochemicall characteristics of women with breast cancer were examined between these two groups. In the first comparison, the patients' ages were compared between the two groups, and there was no difference between the two groups. Furthermore, there was no significant relationship between body mass index and tumor size between the two groups. In addition, the number and percentage of each variable were observed, and the relationship between these variables and their positive or negative in the two groups using the chi-square test or Fisher's exact test were analyzed at a significance level of 0.05. Therefore, it can be suggested that there was no significant relationship between the body mass index and variables in the two groups.

Data relating to the biochemical tests are shown in Table 2. According to the Kolmogorov-Smirnov test, the serum levels of AST and ALT were normally distributed and compared using an independent t-test. Other variables were analyzed using the Mann–Whitney test. According to the results, the serum levels of bilirubin and uric acid had different means in the groups, and the assumption of mean inequality was accepted in the two groups.

### 3.2. Hematological Parameters

Table 3 shows some of the variables in the study groups and a comparison between them using an independent *t*-test and Mann–Whitney test. According to Kolmogorov-Smirnov test, the two variables WBC and MCHC had a normal distribution and were analyzed using an independent t-test, while the other variables did not have a normal distribution. The Mann–Whitney distribution was used to compare the means between two groups. According to the result, the *p* values of HB, HCT, MCV, and MCH were less than 0.05, showing that there were significant differences between the patient and control groups.

### 3.3. Oxidative Stress

**Figure 1:**
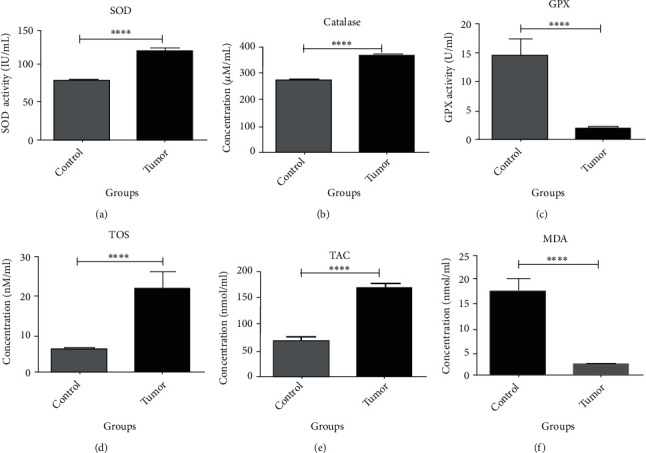
Comparsion of oxidative stress parameters beween cases and controls.

As shown in Table 4 and Figure 1 show, the serum levels of SOD was 117.7 ± 27.44 and 77.88 ± 15.32 in patients with breast cancer and control subjects, respectively, with statistically significant differences between them (*p* value < 0/0005). The serum level of CAT was 369 ± 38.57 and 271.5 ± 51.43 in patients with breast cancer and controls, respectively, without statistically significant differences between them (*p* value = 0.062). The serum levels of GPX were 1.694 ± 2.185 and 14.5 ± 13.35 in patients with breast cancer and controls, with statistically significant differences between them (*p* value < 0/0001). Also, the serum levels of TOS were 22.59 ± 12.46 and 6.462 ± 2.951 in patients with breast cancer and controls, respectively, with statistically significant differences between them (*p* value < 0/0001). The serum levels of TAC in patients with breast cancer and controls were 170 ± 71.97 and 61.03 ± 38.42, respectively, with statistically significant differences between them (*p* value < 0/0002).

Table 5 shows the descriptive oxidative stress index (*OSI*) values (TOS/TAC ratio) for the control and intervention groups. The lowest OSI values are -0.01 in the control group and 0.04 in the patients, and the highest OSI values are 0.43 and 0.42, respectively. Finally, the comparison of means between the two groups was not statistically different. The serum levels of MDA were 8.288 ± 0.0995 and 18.23 ± 9.807 in patients with breast cancer and controls, respectively, with a statistically significant difference between them (*p* value < 0/0001).

### 3.4. Correlation between Oxidative Stress Parameters and Hematological Indices

In this study, the correlation between SOD levels and other variables was determined. As shown in Table 6, HCT was weakly correlated with increased SOD activity (*p* = 0.028; *R* = 0.245). None of the other hematological indices correlated with SOD activity. In addition, the relationship between MDA activity and hematological markers was analyzed and none of the correlations between the variables were significant. Regarding the relationship between serum levels of TOS and hematological parameters, the results showed that although there was a significant correlation between MCV, MCH, and TOS (*p* < 0.05), the correlation coefficient values were 0.262 and 0.255, respectively, indicating a direct and weak correlation. Furthermore, the results showed that there are no significant correlations between serum TAC level and hematological parameters. In addition, according to the results presented in Table 6, while there was a significant correlation between serum GPX activity and hematological parameters (HCT and MCV), considering the correlation coefficient, which was -0.226 and -0.260, respectively, the correlation was inverse and weak (as one of the two variables increased, the other variable decreased).

As shown in Table 6, there was a significant relationship between serum CAT activity and HCT, MCV, and MCH; however, given that the correlation coefficients were 0.239, 0.265, and 0.237, respectively, this direct correlation was weak (when one increases from two variables, the other variable increases).

## 4. Discussion

The main challenge of this study was to evaluate oxidative stress parameters and their correlation with hematological indices in patients with breast cancer. According to the results, serum SOD and CAT levels were higher in the patient group than in the control group. These results are consistent with a report by Rajneesh et al., who found higher SOD and CAT levels in breast cancer patients than in controls [13]. These results are consistent with those reported by Seth et al. [14] and Zińczuk et al. [15], who found that the levels of serum SOD (*p* < 0.0001) and CAT (*p* < 0.0001) were significantly increased in breast cancer patients as compared to controls. This result is contrary to that of Negahdar et al., who found that the rate of SOD and CAT activity in breast cancer patients was significantly lower than that in controls [16]. This inconsistency may be due to an adaptive response to overproduction of intracellular ROS in cancer cells. Increased generation of O_2_ and H_2_O_2_ can induce SOD and CAT activities. Increased superoxide dismutase activity in inflammatory cells leads to increased production of hydrogen peroxide. Pietarinen-Runti et al. observed that among all inflammatory cells, neutrophils had the highest CAT activity [17]. Although the gene expression of SOD was not evaluated in this study, based on the findings of previous studies, increased SOD mRNA expression was observed in patients with breast cancer [18].

Regarding GPX activity in patients with breast cancer, the results of our study showed a lower level of serum GPX in the patient group than in the control group. This finding was consistent with that of Kangari et al. who reported decreased GPX activity in breast tumors [19]. Reduced GPX activity may lead to the accumulation of reactive hydrogen peroxide. We studied serum oxidant stress indices that may be influenced by other related factors in the blood. One study reported that GPx levels were inversely correlated with blood pressure, and, only GPx was influenced by the number of metabolic syndrome (MetS) components [20]. However, elevated GPX activity has also been reported in breast cancer [21]. These results may be explained by the fact that patients in the initial stages have high oxidative stress and lipid peroxidation. The level of free radicals may be higher, and the body tries to compensate for this by increasing the level of antioxidants. Thus, the increased serum activity of antioxidants might be the result of a natural defense mechanism to fight carcinogenesis. It is suggested that the increased activity of CuZn-SOD leads to an increase in the conversion of O_2_^·^ – into H_2_O_2_; however, H_2_O_2_ cannot be detoxified because of the decreased activity of GPx.

MDA is formed following the peroxidation of unsaturated fatty acids by an enzymatic reaction in which free radicals damage unsaturated fatty acids as the main constituents of cell membranes. Therefore, serum MDA level is an indicator of lipid peroxidation and a suitable noninvasive biomarker in the oxidative stress assay [22]. Several studies have shown an increased MDA concentration in patients with breast cancer compared with that in control individuals [23–26]. One unexpected finding was the lower MDA concentration in the patient group than that in the control group. In accordance with the present results, a previous study showed that MDA levels were significantly lower in gastric and colorectal cancer patients than in those of controls. It was difficult to explain the reduced MDA levels, but it might be related to the tumor aggressiveness behavior in a way that oxidant-antioxidant status is favorable for the growth of rapidly dividing cells. Rapidly proliferating tumor cells are resistant to lipid peroxidation and overexpress antioxidant enzymes [27]. According to Didžiapetrienė et al., the changes in the MDA level are dependent on the age of patients and the stage of disease [28]. In addition, we studied serum oxidant stress indices that may be influenced by other related factors. In general, it can be justified that oxidant and antioxidant enzymes in the blood are affected by various factors, including inflammatory factors such as IL-6 and blood sugar levels [29]. In addition, oxidative damage to lipids is related to physiologic conditions such as increasing levels of body fat and cholesterol, smoking, and inflammation (reflected in C-reactive protein), which may increase with arthritis and other conditions of aging [30].

TAC states the enzymatic and nonenzymatic antioxidants, while TOS describes the oxidants contained in a sample. OSI (TOS/TAC ratio) designates the correlation between antioxidant and oxidant concentrations. One of the significant findings of our study was that TOS and TAC levels were higher in the patient group than in the control group. In accordance with the present results, Nsonwu-Anyanwu et al. reported higher TAC levels in malignant breast cancer than the controls [31]. The levels observed in this study were far below those observed by Şener et al. [32]. The higher TAC levels may represent a compensatory up regulation of antioxidant activities in response to increased lipid peroxidation and oxidative stress to maintain new redox equilibrium and confer resistance in the cells against oxidative insult observed in this group. In addition, the opposite results may be due to the levels of oxidative stress markers in blood samples, which are different from those in breast tissue samples. Our experiments are in line with previous results by Bayhan et al. [33], Youssef and Salem [34], and Yang et al. [35].

The complete blood cell count (CBC) is low-cost, standardized, and reliable prognostic factor in cancer. For example, a 2015 study showed that a decrease in absolute leukocytes number was associated with an increase in the breast cancer stage [36]. The white blood cell (WBC) count has been used as a marker of infection and inflammation. As in our study, Margolis et al. showed that women with higher WBC counts have an increased risk of developing invasive breast cancer [37].

There are approximately 2016 reports of the use of the CBC test to diagnose breast cancer. In the present study which was performed on 162 patients with breast cancer, the relationship between red blood cell count (RCI) indicators, such as RBC, HCT, MCHC, MCV, MCH, and neutrophil-to-lymphocyte ratio (NLR) in CBC tests with tumor size, clinical stage of the disease, and tumor histology has been studied. Disease-free survival analyses showed that patients with higher mean hemoglobin (MCH) levels had shorter disease-free survival than those with low MCH levels. It can be concluded that the measurement of MCH can be used as a predictable indicator in direct correlation with disease-free survival in patients with breast cancer [38]. Cui et al. showed that RBC, HGB, HCT, MCH, MCHC, and RDW ratios before and after treatment were not associated with outcomes [39]. In contrast to earlier findings, we showed that there were significant differences in Hb, HCT, MCV, and MCHC between patients with breast cancer and healthy subjects. This study supports the clinical observations of Divsalar et al., who reported a significant (*p* < 0.05) difference in Hb, HCT, and MCV parameters between breast cancer patients and control group [40]. A possible explanation for this might be the differences in the sample size, disease stage, the tumor size, and the treatment type of the patients.

In the current study, the next step was to determine the link between oxidative stress parameters and hematological indices. We found that HCT poorly correlated with increased SOD activity. In line with our findings, Asmah et al. showed that HCT is positively and significantly related to SOD activity [41]. Our observations are inconsistent with those of Asmah et al., who studied patients with diabetes. There was not significant difference between SOD and HTC levels [42]. Hypoglycemic agents such as insulin can affect oxidative stress, and this difference may be due to the differences in the studied groups. In the present study, we found no correlation between MDA levels and hematological parameters in patients with breast cancer. This outcome is contrary to that of Basavaraj who found that serum MDA levels alter hematological indices in patients with breast cancer [43]. It can be concluded that the difference could be due to the differences in the studied patients. In our study, patients did not receive any treatment; however, in their study, patients underwent chemotherapy. In conclusion, oxidative stress appears to be positively and negatively correlated with several hematological parameters. Our findings also suggest the possible use of these parameters to screen and monitor breast cancers.

## 5. Conclusion

In conclusion, the present study revealed a significant difference in oxidative stress between breast cancer patients and healthy individuals and a correlation between oxidative stress parameters and hematological parameters. There were some limitations to this study, including the small sample size. In addition, this study could not completely define the cause and effect relationship of the parameters. Further, larger prospective studies are necessary to explain the biological mechanisms of this relationship.

## Figures and Tables

**Table 1:** Patient characteristics.

Characteristics		Cases BMI < 30 kg/m^2^	Cases BMI ≥ 30 kg/m^2^	*p* value
Age ± SD (years)		51.45 (11.81)	55.83 (11.97)	0.236

Tumor size	≤20 *n* (%)	9 (45%)	9 (39.1%)	0.763
	>20 *n* (%)	11 (55%)	14 (60.9%)	

Lymph nodes	Positive n (%)	12 (60.0%)	9 (39.1%)	0.172
	Negative *n* (%)	8 (40.0%)	14 (60.9%)	

Histology	Ductal *n* (%)	18 (90.0%)	22 (95.7%)	0.590
	Lobular *n* (%)	2 (10.0%)	1 (4.3%)	

ER	Positive *n* (%)	15 (75.0%)	19 (82.6%)	0.711
	Negative *n* (%)	5 (25.0%)	4 (17.4%)	

PR	Positive *n* (%)	14 (70.0%)	17 (73.9%)	0.755
	Negative *n* (%)	6 (30.0%)	6 (26.1%)	

HER2	Positive *n* (%)	3 (15.0%)	5 (21.7%)	0.704
	Negative *n* (%)	17 (85.0%)	18 (78.3%)	


Comparison between the variables in the subjects studied that these individuals were divided into two groups according to the value of body mass index. The criterion for dividing the two groups was body mass index above 30 and less than 30 kg/m^2^.

**Table 2:** Biochemical functions in study groups (mean ± SD).

Variables	Cases	Controls	*p* value
AST	23.60 (7.93)	22.38 (8.35)	0.503
ALT	22.95 (7.94)	21.84 (7.01)	0.511
Bilirubin	0.45 (0.23)	0.84 (.26)	<0.001^∗^
Uric acid	5.10 (0.94)	4.54 (0.73)	<0.001^∗^

^∗^ *p* value < 0.05 is considered significant.

**Table 3:** Hematologic indices in patients with breast cancer and healthy subjects.

Parameters	Cases	Controls	*p* value
WBC (/*μ*L)	7339.53 (1791.28)	7208.11 (1726.69)	0.740
RBC (/*μ*L)	5855.58 (5838.02)	4751.89 (1150.54)	0.262
Hb (g/dL)	14.04 (2.40)	13.04 (2.19)	0.011^∗^
HCT (%)	42.96 (6.20)	39.04 (6.58)	0.001^∗^
PLT (10^3^/*μ*L)	272.09 (84.50)	268.51 (77.39)	0.675
MCV (fL)	87.75 (14.63)	81.51 (11.02)	0.016^∗^
MCH (pg)	29.92 (4.15)	27.63 (4.72)	0.023^∗^
MCHC (g/dL)	33.32 (1.94)	32.97 (1.718)	0396

WBC: white blood cells; RBC: red blood cells; Hb: hemoglobin; HCT: hematocrit; PLT: platelets; MCV: mean corpuscular volume; MCH: mean corpuscular hemoglobin or mean cell hemoglobin; MCHC: mean corpuscular hemoglobin concentration. ^∗^*p* value < 0.05 is considered significant.

**Table 4:** Comparison oxidative stress parameters between cases and controls.

Parameters	Cases	Controls	*R* square	*p* value
Total SOD activity (U/mL)	117.7 ± 27.44	77.88 ± 15.32	0.4341	0.0005^∗∗∗^
Total CAT concentration (m/mL)	369 ± 38.57	271.5 ± 51.43	0.5465	0.062
Total GPX activity (U/mL)	1.694 ± 2.185	14.5 ± 13.35	0.3302	0.0001^∗∗∗^
Total TOS concentration (m/mL)	22.59 ± 12.46	6.462 ± 2.951	0.431	0.0001^∗∗∗^
Total TAC concentration (m/mL)	170 ± 71.97	61.03 ± 38.42	0.4659	0.0002^∗∗∗^
Total MDA concentration (m/mL)	8.288 ± 0.995	18.23 ± 9.807	0.362	0.0001^∗∗∗^

**Table 5:** Comparison of OSI between case and control groups.

Subjects	*N*	Minimum	Maximum	Mean	Std. deviation	*p* value
Case	43	0.04	0.43	0.1572	0.09598	0.775
Control	37	-0.01	0.42	0.1506	0.11240	

**Table 6:** Correlation between oxidative stress parameters and haematological indices.

	SOD		MDA		TOS		TAC		GPX		CAT	
Pearson's	*R*	sig	*R*	sig	*R*	sig	*R*	sig	*R*	sig	*R*	sig
WBC	-0.038	0.740	-0.111	0.325	-0.045	0.692	0.080	0.478	0.003	0.978	.029	.800
RBC	0.073	0.518	-0.027	0.812	-0.046	0.685	-0.049	0.665	-0.089	0.431	.156	.166
HGB	0.217	0.054	-0.060	0.598	0.183	0.105	0.111	0.326	-0.146	0.197	.183	.105
HCT	0.245^∗^	0.028	-0.078	0.489	0.207	0.066	0.226	0.044	-0.226^∗^	0.044	.239^∗^	.033
PLT	0.015	0.893	-0.044	0.696	-0.091	0.423	0.081	0.475	-0.036	0.754	-.037	.745
MCV	0.203	0.070	-0.117	0.300	0.262^∗^	0.019	0.148	0.191	-0.260^∗^	0.020	.265^∗^	.017
MCH	0.124	0.275	0.093	0.410	0.255^∗^	0.022	0.194	0.085	-0.201	0.074	0.237^∗^	0.034
MCHC	0.076	0.503	-0.039	0.731	0.073	0.518	0.093	0.414	0.017	0.881	0.112	0.323

## Data Availability

All data used and analyzed during the current study are included in this manuscript and available from the corresponding author on reasonable request.
